# Tea plant genomics: achievements, challenges and perspectives

**DOI:** 10.1038/s41438-019-0225-4

**Published:** 2020-01-01

**Authors:** En-Hua Xia, Wei Tong, Qiong Wu, Shu Wei, Jian Zhao, Zheng-Zhu Zhang, Chao-Ling Wei, Xiao-Chun Wan

**Affiliations:** State Key Laboratory of Tea Plant Biology and Utilization, Anhui Agricultural University, Hefei, 230036 China

**Keywords:** Genomics, Plant genetics

## Abstract

Tea is among the world’s most widely consumed non-alcoholic beverages and possesses enormous economic, health, and cultural values. It is produced from the cured leaves of tea plants, which are important evergreen crops globally cultivated in over 50 countries. Along with recent innovations and advances in biotechnologies, great progress in tea plant genomics and genetics has been achieved, which has facilitated our understanding of the molecular mechanisms of tea quality and the evolution of the tea plant genome. In this review, we briefly summarize the achievements of the past two decades, which primarily include diverse genome and transcriptome sequencing projects, gene discovery and regulation studies, investigation of the epigenetics and noncoding RNAs, origin and domestication, phylogenetics and germplasm utilization of tea plant as well as newly developed tools/platforms. We also present perspectives and possible challenges for future functional genomic studies that will contribute to the acceleration of breeding programs in tea plants.

## Introduction

The tea plant (*Camellia sinensis*) is an economic crop with significant importance^1^. Its leaves are generally used to produce tea—the world’s most popular non-alcoholic beverage. Tea comprises abundant characteristic compounds, such as tea polyphenols, theanine, and caffeine, which not only determine the quality of tea but also confer it with tremendous health benefits^2^. However, compared to other crops, such as rice^3^, research on the functional genomics of tea plants is lagging behind, which has hampered the utilization of genetic resources for modern molecular breeding, which is urgently needed. The rapid development and innovation of biotechnologies, particularly next-generation sequencing (NGS), has resulted in significant progress in understanding the genomics and genetics of tea plants in the past two decades, which can be briefly summarized according to five aspects: (1) the deciphering of the genome and transcriptome of tea plants using diverse NGS technologies; (2) the identification and functional analysis of tea quality-related genes and their regulatory networks; (3) the investigation of the epigenetics and noncoding RNAs (ncRNAs) involved in stress responses and other biological processes; (4) the exploration of the diversification and domestication of tea plants; and (5) the development of innovative tools and techniques for the characterization of novel genes and alleles. In this review, we briefly summarize the main achievements of the last two decades and present potential challenges and perspectives for future functional genomic studies that would contribute to the acceleration of breeding programs in tea plants.

## Whole-genome sequencing of tea plants

### Cytogenetics of tea plants

Tea plants are an economically important crop. Extensive investigations of tea plant karyotypes have facilitated our fundamental understanding of their chromosomal biology^4–7^. The first report of the chromosome karyotype analysis of tea plants can be traced back to the last century. Morinaga and colleagues observed that 15 chromosomes (*n* = 15) were present in the gametes of *C. sinensis* tea plants and that 30 chromosomes (2*n* = 30) occurred in the zygotes of *C. japonica*, a closely related species of tea plant^8,9^. These findings suggested that the diploid tea plant has 30 chromosomes and that the chromosome number might be conserved in genus *Camellia*. New innovations in biotechnologies gradually confirmed these conclusions among globally collected tea clones^5,7,10–12^. In particular, the investigation of the chromosome numbers and ploidy levels of 139 tea individuals representing almost all sections of genus *Camellia* revealed the diploid nature of the majority (90.65%) of *Camellia* species, with a chromosome number of 2*n* = 30^6^. Only a few tea plant species are polyploid, which exhibit 45–120 chromosomes. These species predominantly come from section *Camellia* (*C. chekiangoleosa*, *C. mairei*, and *C. reticulata*), section *Oleifera* (*C. oleifera* and *C. sasanqua*), and section *Archecamellia* (*C. granthamiana*).

Genome size refers to the DNA content within the haploid genome of an organism. The estimation of genome size is not only of great significance for cytogenetic studies but also provides indispensable basic data for genome sequencing, comparative genomics, and evolutionary analyses. The genome size of tea plants was initially estimated to be 3.8–4.0 Gb^13,14^, which was further demonstrated by the investigation of 36 Indian tea accessions, where all the investigated accessions exhibited diploid genomes with sizes ranging from 3.5 to 3.8 Gb^15^. The recent genome sequencing of two representative elite tea plant cultivars, *C. sinensis* var. *sinensis* cv. shuchazao and *C. sinensis* var. *assamica* cv. yunkang#10, showed an ~3.0 Gb genome size, which is similar to that of maize but much larger than those of coffee and cocoa^1,2^. While genome size is likely to be conserved in cultivated tea plants, intraspecific and interspecific variations are also observed in genus *Camellia*, possibly due to the frequent hybridization and polyploidization events that occur after speciation^6^.

### Genetic linkage maps of tea plants

The construction of genetic linkage maps is the basis of molecular biology and is essential for a wide range of genetics and genomic studies, such as quantitative trait mapping, molecular marker-assisted breeding and comparative genomic studies^16^. Unlike other crops, tea plants are self-incompatible species with relatively low cross-fertility rates, resulting in a lack of a high generation segregation populations and sufficient offspring for genetic map construction^17^. More than 10 genetic linkage maps have been generated for tea plants, most of which are produced based on an F1 population consisting of <150 offspring^18–22^ (Table 1). The dominant molecular markers used for genetic map construction include random amplification of polymorphic DNA (RAPD) markers^18,20,23–26^, amplified fragment length polymorphism (AFLPs)^18–20^, inter-simple sequence repeats (ISSRs)^24^, simple sequence repeats (SSRs)^20,25–28^, and single nucleotide polymorphisms (SNPs)^21,29^. These reported genetic maps vary in length and density, with the total map length ranging from 1180.9 to 3314.3 cM and the average distance between adjacent markers from 0.4 to 20.1 cM. Such differences are principally affected by the total number of individuals and types of molecular markers applied for map construction. The highest-density genetic map of tea plants obtained to date was constructed using a total of 4217 polymorphic SNP markers developed from 327 individuals of the F1 segregating population (LJ43 × BHZ) via 2b-RAD sequencing^29^. The resultant genetic maps consisted of 15 linkage groups ranging from 87.46 to 146.93 cM. The total map length was 1679 cM, with an average interval of 0.4 cM between 4217 adjacent markers. LG01 and LG03 were the two largest linkage groups, with genetic distances of 146.93 cM (374 markers) and 146.61 cM (374 markers), respectively. Despite the numerous genetic maps currently available, the density and quality of these maps need to be further improved by investigating larger populations and applying more advanced biotechnologies. The resultant data will be important for future investigations of tea plant biology that will benefit the whole tea industry.

**Table 1:** Summary of the representative linkage maps of tea plants.

Mapping population	Size^a^	Marker type	No. of markers	Total length (cM)	Marker density (cM)	No. of linkage groups	Reference
Sayamakaori × Kana-CK17	54	RAPD	140	1640	11.7	17	Ota et al.^23^
SFS150 × TN14/3	90	RAPD AFLP	126	1350	11.7	15	Hackett et al.^18^
Keemun #4 × Chaoandawuye	69	AFLP	208	2458	11.9	17	Huang et al.^19^
Fudingdabaicha × Zhenong #12	94	ISSR RAPD	62	1181	20.1	7	Huang et al.^24^
SFS150 × S15/10	42	SSR RAPD AFLP	100	1412	14.1	30	Kamunya et al.^20^
Sayamakaori × Kana-CK17	54	SSR STS CAPS RAPD	279	1218	4.35	15	Taniguchi et al.^25^
Yingshuang × Beiyuedanzhu	183	SSR	406	1144	2.9	15	Ma et al.^27^
Yingshuang × Beiyuedanzhu	148	SSR SNP	6448	3965	1.0	15	Ma et al.^21^
Long #43 × Baihaozao	170	SSR	483	1226	2.5	15	Tan et al.^28^
Fushun × Kemsull	79	SSR RAPD AFLP	678	1442	4.7	15	Chang et al.^26^
Long #43 × Baihaozao	327	SNP	4217	1679	0.4	15	Xu et al.^29^

^a^Population size

### Current progress in tea plant genome sequencing

**Fig. 1:**
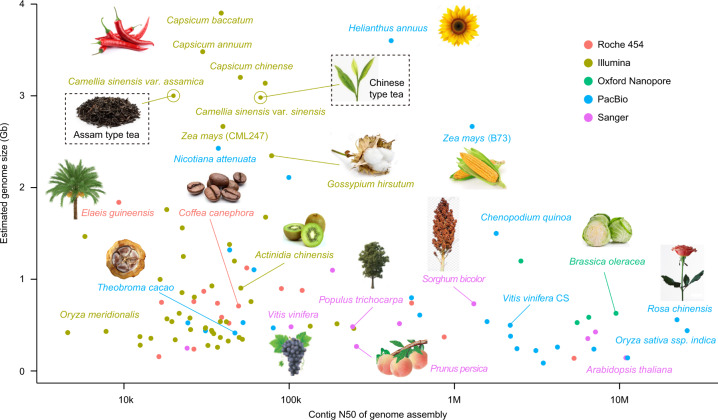
Current genome sequencing progress in tea and other plants. The *x*-axis represents the contig N50 of the genome assembly, while the *y*-axis shows the estimated genome size of each plant. The sequencing platforms are indicated in red (Roche 454), brown (Illumina), green (Oxford nanopore), blue (PacBio SMRT), and pink (Sanger). Tea plants are highlighted with a rectangular box.

With the revolution of sequencing technology, an increasing number of labs around the world have successfully released more than 236 plant genomes, and the tea plant genome is probably among the most complex of these^30^ (Fig. 1). This is chiefly because tea plants have an extremely large, highly heterozygous, repeat-rich nuclear genome, presenting major challenges in genome assembly. The tea community has put forth its best effort to assemble and release the genomic sequences of two primary tea plant varieties: *C. sinensis* var. *sinensis* (CSS; Chinese type tea) and *C. sinensis* var. *assamica* (CSA; Assam type tea), using NGS technologies^1,2^. Both of the assemblies are composed of ~3 Gb of genomic sequences with an average scaffold N50 size of 920 Kb, which is much larger than those from other plants with complex genomes, such as the orchid (scaffold N50 = 359 Kb)^31^ and moso bamboo (scaffold N50 = 329 Kb)^32^. Nevertheless, compared to other model species, such as rice^33^, the scaffold N50 sizes of the current assemblies are still somewhat disappointing, due largely to the limitation imposed by the short read length generated from NGS technology (Fig. 1). Unassembled regions account for ~5% of the current assemblies. In the future, additional efforts will be needed to further improve the quality and completeness of the genome assemblies of tea plants, particularly with the upcoming advances in sequencing technologies (e.g., 3rd-generation sequencing) and new algorithm-derived assemblers.

There are 33,932 and 36,951 protein-coding genes in the CSS and CSA tea plant genomes, respectively. Transposable elements (TEs) account for 64% and 80% of the CSS and CSA genomes, respectively. LTR retrotransposons are among the most dominant TEs, independently representing 58% and 67% of the CSS and CSA genomes, respectively. The rapid propagation of TEs, particularly the proliferation and persistence of a single Ty3/gypsy retrotransposon family (*TL001*) for over 50 million years, has been proven to have led to a considerable increase in genome size in tea plants^1^. CSA and CSS diverged from their common ancestor ~0.38–1.54 million years ago (mya). However, this divergence time might have been underestimated because of the draft nature of the two current genome assemblies, which generated only a small proportion of collinear genes for dating. Similar to other plants, tea plants underwent two whole-genome duplication (WGD) events, including recent and ancient events occurring 30–40 and 90–100 mya, respectively^2^.

Tea plants contain rich and diverse secondary metabolites that not only confer tea quality characteristics, such as color, aroma and taste, but also play critical roles in the responses to biotic and abiotic stresses^34^. Several genes encoding enzymes associated with the biosynthesis of secondary metabolites were significantly amplified in the tea plant genome. In particular, the striking expansion of serine carboxypeptidase-like (SCPL) acyltransferase-encoding genes might directly contribute to the high accumulation of galloylated catechins that determine tea palatability^2^. Compared to kiwifruit, tomato, potato, cacao, and *Arabidopsis thaliana*, tea plants show expansion of disease resistance genes, including nucleotide-binding sites with leucine-rich repeats (NBS-LRRs) and pattern-recognition receptors (RLK-LRRs)^1^. This contributes to the enhancement of the immune system of tea plants and, thus, their adaptation to diverse global environments. WGD events and subsequent tandem duplications are the two major evolutionary forces that drive such gene expansions.

Tea-processing suitability refers to the characteristics of tea varieties that make them suitable for the manufacture of certain types of tea to achieve the best quality. It is accepted that different varieties of tea plants differ in terms of flavors and tastes. The sequencing of the tea plant genome together with the transcriptomes and metabolomes of 25 *Camellia* species representing almost all the sections from genus *Camellia* showed that, despite the conservation of three metabolic pathways (catechins, theanine, and caffeine) among *Camellia* species, most of the flavonoid and caffeine, but not the theanine-related genes, exhibited higher expression levels in species from section *Thea* than in non-*Thea* species^1^. This elucidates why the leaves of tea plants from non-*Thea* sections, such as well-known ornamental camellias (e.g., *C. japonica*) and the traditional oil tea plant (*C. oleifera*), accumulate such low contents of catechins and caffeine but not theanine, making them unsuitable for tea production. Further investigation of the roles of natural selection and artificial selection in shaping the genes involved in the three metabolic pathways will contribute to a deep understanding of the processing suitability and quality of tea. In addition, caffeine (1,3,7-trimethylxanthine) is among the most well-known purine alkaloids in plants, and its biosynthesis is mainly catalyzed by the N-methyltransferases (NMTs)^35^. The *NMT* genes have undergone recent rapid and independent evolution in tea plants relative to cocoa and coffee^1^.

### Organelle genome sequencing

Plants generally harbor two independent organelle genomes (chloroplast and mitochondria), which provide invaluable resources for a range of functional, evolutionary and comparative genomic studies^36^. The cultivated tea plant harbors a circular chloroplast (cp) genome of 157,096 bp in length with an overall GC content of 37.3%^37^. It exhibits the typical plant cp genome structure, including a pair of inverted repeat regions (IRs, 26,080 bp) separated by a large single-copy region (LSC, 86,653 bp) and a small single-copy region (SSC, 18,283 bp). The whole-genome sequence encodes a total of 133 cp genes, 86 of which are protein-coding genes, while 8 are rRNA genes, and 39 are tRNA genes. The cp genomes have been assembled for diverse well-known tea cultivars, such as “Longjing #43”^37^, “Yunkang #10”^38^, and “Shuchazao”^2^. In addition to cultivated tea plants, an increasing number of cp genomes from species of genus *Camellia* have also been released, including those of *C. japonica* (NC_036830), *C. mairei* (NC_035688), *C. szechuanensis* (NC_035651), *C. elongata* (NC_035652), *C. azalea* (NC_035574), *C. pubicosta* (NC_024662), *C. petelotii* (NC_024661), *C. reticulata* (NC_024663), *C. grandibracteata* (NC_024659), *C. leptophylla* (NC_024660), *C. crapnelliana* (NC_024541), *C. oleifera* (NC_023084), *C. danzaiensis* (NC_022460), *C. impressinervis* (NC_022461), *C. yunnanensis* (NC_022463), *C. cuspidata* (NC_022459), *C. pitardii* (NC_022462), and *C. taliensis* (NC_022264)^39–45^. The *Camellia* cp genomes possess quite similar genome sizes, total numbers of genes, and sequence homology, suggesting extreme genome conservation during evolution.

In sharp contrast to the chloroplast genome, only a partially assembled mitochondrial (mt) genome is available for cultivated tea plants^38^. The current release of the tea plant mt genome consists of two circular scaffolds with total lengths of 702,253 bp (45.63% GC) and 178,082 bp (45.81% GC). It encodes 71 mt genes, including 44 protein-coding genes, 24 tRNA genes, and 3 rRNA genes. Compared to the cp genome, the tea plant mt genome shows a repeat-rich nature, including a total of 38,027 bp of long repeat sequences^38^.

## Transcriptome sequencing and gene discovery in tea plants

### Transcriptome sequencing of tea plants

Transcriptome sequencing has revolutionized genetic and functional genomic studies of organisms, particularly for nonmodel species without available sequenced genomes^46^. In the last decade, innovations overcoming challenges in tea plant genome sequencing have greatly accelerated the transcriptomic investigation of this economically important crop. Shi and colleagues examined the first transcriptome of tea plants (cultivar shuchazao) using Illumina sequencing technology and obtained a total of 127,094 unigenes, which were applied for the in-depth exploration of candidate genes involved in the biosynthesis of characteristic compounds of tea plants that determine tea quality^47^. Subsequently, growing transcriptome-sequencing projects were launched to further investigate the gene expression dynamics of tea plants under cold acclimation^48–51^, drought stress^52,53^, and hormone responses^54^ and the mechanisms underlying self-incompatibility^17,55^, nitrogen utilization^56,57^, trichome formation^58^, and tea quality^59–62^. These results greatly broadened our understanding of tea plant biology. With the advent of single-molecule sequencing technology, a recent study produced a more accurate full-length transcriptome of tea plants^63^. In addition to the cultivated tea plants, several transcriptomes from closely related species from genus *Camellia* have also been reported, which have provided indispensable resources for comparative transcriptomic studies^64–66^ (Supplementary Table 1). The sequencing of the transcriptome of oil tea plants (*C. oleifera*) identified 3022 orthologous gene pairs between cultivated tea plants and oil tea plants, among which 211 exhibited evidence of positive selection^64^. Compared to the cultivated tea plants, *C. taliensis* showed extraordinary amplification of cold tolerance-related genes^65^. Most of the genes associated with triacylglycerol biosynthesis in *C. reticulata* and *C. sinensis* are multiple-copy genes, suggesting the potential occurrence of WGD events in the common ancestor of genus *Camellia*^66^. An increasing number of transcriptome-sequencing projects have also been carried out in other *Camellia* species, such as *C. sasanqua*^67^, *C. chekiangoleosa*^68^, and *C. nitidissima*^69^. These datasets expanded the gene pool of tea plants and will be of particular importance for future tea plant breeding, as well as investigations of functional genomics, phylogenomics, and comparative transcriptomics. It should be noted that the current reported transcriptomes from both tea plants and other *Camellia* species vary in the total number of assembled transcripts and N50 sizes, primarily due to the different sequencing depths and assemblers adopted. In the future, more efforts should be made to evaluate the optimized-sequencing depth and assembler to better construct the transcriptomes of tea plants.

### Functional gene sets and their regulatory networks

One fundamental mission of the molecular biology research on tea plants is to understand the functions and regulatory mechanisms of genes encoded by the genome. During recent decades, rapid advances in biotechnologies have facilitated the cloning and functional characterization of an increasing number of genes in tea plants^70–72^. Most of these genes have been identified from CSS and CSA, in which the numbers of cloned genes are much greater than those from other closely related tea plant species. Their functions can generally be classified into three major categories, including secondary metabolite biosynthesis^73–80^, abiotic and biotic stress responses^81–86^, and aroma formation^70,71,81,87–91^ (Table 2). Among these genes, those associated with secondary metabolite biosynthesis and aroma formation are the most studied because they directly determine the quality of tea. Supplementary Table 2 summarizes the details of the currently available cloned genes in tea plants. We here selectively highlight some representatives that would be preferred candidates for the future genetic improvement of tea plants.

**Table 2:** List of representative functional genes in tea plants.

Gene symbol	NCBI accession	Length (bp)	Function description	Ref.
*Polyphenol biosynthesis*				
*CsANR1*	GU992402	1044	Encoding anthocyanidin reductase	^77^
*CsANR2*	GU992400	1014	Encoding anthocyanidin reductase	^77^
*CsLAR*	GU992401	1282	Involved in flavan-3-ol biosynthesis	^77^
*CsF3'H*	KT180309	1706	Encoding flavonoid 3'-hydroxylase	^92^
*CsC4H*	KY615675	1518	Encoding cinnamate 4-hydroxylase in synthesis of flavonoids and lignins	^93^
*CsF3H*	KY615688	1107	Encoding flavanone-3-hydroxylase	^94^
*CsMYB4a*	KY774676	735	Negatively regulates phenylpropanoid and shikimate pathway	^78^
*CsMYB5*	KY827396	930	Regulate anthocyanin and proanthocyanidin biosynthesis	^79^
*CsMYB75*	GGTM01017033	765	Regulate anthocyanin hyperaccumulation	^76^
*CsGST*	MK431867	642	Encoding glutathione-S-transferase	^95^
*Caffeine biosynthesis*				
*CsTCS*	AB031280	1438	Caffeine synthase gene	^73^
*Theanine biosynthesis*				
*CsTSI*	TEA015198^a^	2544	Theanine synthetase gene	^2^
*CsAAPs*	TEA031577^a^	2339	Encoding amino acid permease involved in theanine transportation	^96^
*Aroma formation*				
*Csβ-PD*	AB088027	1729	Encoding beta-primeverosidase in floral aroma formation	^88^
*CsGT1*	AB847092	1458	Converting volatile organic compounds into β-primeverosides for aroma biosynthesis	^90^
*CsSAMT*	MG459470	1104	Encoding the salicylic acid carboxyl methyltransferase for methyl salicylate formation	^97^
*CsLIS/NES*	KF006849	2050	Producing (E)-nerolidol and linalool in vitro	^87^
*CsUGT85A53*	NA	1455	Formation of (Z)‐3‐hexenyl glucoside, novel insect pest control	^81^
*Abiotic and biotic stress*				
*CsCOR1*	EU563236	755	Involved in cold, salinity and dehydration tolerance	^82^
*CsHSP17.2*	KU244518	734	Mediates tea plant thermo tolerance	^98^
*CsICE1*	GQ229032	1964	Inducer of CBF expression	^84^
*CsCBF1*	EU563238	1210	C-repeat-binding factor	^84^
*CsTLP*	DQ444296	681	Increases tolerance to fungal pathogens overexpressed in potato	^83^
*CsGolS2*	KP757767	1574	Response to *Ectropic oblique* attack	^86^

^a^CSS locus ID (from TPIA: http://tpia.teaplant.org)

Caffeine is the most abundant purine alkaloid in the majority of tea plants^99^. It is a crucial component of tea quality and is significantly related to the bitterness of tea. Nevertheless, excessive intake of caffeine has been reported to have some side effects for human health, such as increasing the risk of cardiovascular disease, palpitations, and insomnia^100,101^. The documentation of the genes controlling caffeine biosynthesis is therefore essential for the future of breeding new varieties with a low caffeine content. Kato et al.^73^ cloned the gene coding caffeine synthase (*TCS*) from the young leaves of tea plants using the rapid amplification of complementary DNA ends (RACE) technique^73^. This gene is 1438 bp in size and encodes 369 amino acids. The expression of the *TCS* gene in *Escherichia coli* enables high production of caffeine after feeding on xanthine and S-adenosylmethionine as substrates, confirming the caffeine synthase activity of *TCS*. Theanine is another characteristic amino acid of tea plants and is related to the fresh tastes of tea. The gene encoding enzyme associated with theanine biosynthesis (*CsTSI*) was identified through a combination of genomic and transcriptomic analyses^2^. This gene shares high homology with the glutamine synthetase (*PtGS*) gene of *Pseudomonas taetrolens*, indicating its potential bacterial origin^102^. The overexpression of *CsTSI* in *A. thaliana* significantly increases the accumulation of theanine after ethylamine feeding. Tea polyphenols are the major secondary metabolites of tea plants^103^ and are closely related to the astringent and bitter taste of tea. Two enzymes, UDPglucose:galloyl-1-O-β-d-glucosyltransferase (UGGT) and epicatechin:1-O-galloyl-β-d-glucose O-galloyltransferase (ECGT), were purified and shown to be involved in the biosynthesis of galloylated catechins^74^. Three UDP-glycosyltransferase (*UGT*) genes, *CsUGT84A22*, *CsUGT78A14*, and *CsUGT78A15*, were found to be involved in the biosynthesis of β-glucogallin and glycosylated flavonols^75^. Anthocyanin is another type of polyphenol that typically accumulates in purple tea varieties. It has considerable health benefits and has been used to develop anthocyanin-enriched beverages. The expression of a gene encoding leucoanthocyanidin reductase (CsLAR) in *E. coli* results in the production of 2R,3S-trans-flavan-ol (+)-catechin after the feeding of leucocyanidin as a substrate^77^. Similarly, the expression of two anthocyanidin reductase (CsANR1 and CsANR2)-encoding genes in *E. coli* enables the conversion of cyanidin to a mixture of (+)-epicatechin and (−)-catechin^77^. More recently, *CsMYB75* and *CsGSTF1* were found to be involved in anthocyanin accumulation in purple tea^76^. *CsMYB75* can promote the expression of *CsGSTF1* in transgenic tobacco plants. *CsGSTF1* enables the transfer of anthocyanin from the endoplasmic reticulum (ER) to vacuoles^76^. In tea plants, the regulation of the catechin metabolic pathway is complex, and several transcription factors (e.g., MBW complexes) are involved^76,78–80,104^.

Aroma is vital for tea quality and for attracting global interest. Since the advent of new chemical analytical techniques, such as mass spectrometry, considerable efforts have been made to identify volatile constituents of different types of teas and to assess volatile odor activities and their contribution to tea aroma^70,71,88,89,105–109^. Significant studies on tea aroma biology have revealed that hundreds of volatile terpenoids, in addition to some other volatiles, such as *cis*-3-Hexen-1-ol, are present in tea as glycosides^110–112^, which can be released during the tea manufacturing process^113^. A tea beta-primeverosidase gene was isolated and expressed in *E. coli*. The prokaryotically expressed mature protein was found to be able to hydrolyze beta-primeverosides, liberating a primeverose unit and aglycons and playing roles in tea plant defense and floral aroma formation during the tea manufacturing process^88^. Two UGTs from *C. sinensis*, UGT85K11 (CsGT1) and UGT94P1 (CsGT2), were found to convert volatiles into β-primeverosides via sequential glucosylation and xylosylation, respectively^90^. More recently, a UGT gene (CsUGT85A53) that was functionally validated was shown to enable the conversion of exogenous (Z)-3-hexenol from damaged adjacent tea leaves to its glucoside form, enhancing the ability of tea plants to defend against pests^81^. Studies on tea volatile biosynthesis have also revealed an interesting bifunctional linalool/nerolidol synthase gene (CsLIS/NES) in tea plants^87^. This gene transcribes two transcript isoforms, CsLIS/NES-1 and CsLIS/NES-2, which possess distinct subcellular localizations and molecular functions, with CsLIS/NES-1 localizing to chloroplasts and functioning as linalool synthase, while CsLIS/NES-2 localizes to the cytosol and potentially acts as a nerolidol synthase. In addition, the biosynthesis of the tea volatiles indole, linalool, and nerolidol was found to be controlled by the corresponding genes in tea^89,91,114^. Collectively, the aforementioned genes that were cloned and functionally validated in tea plants have broadened our understanding of the genetic basis of tea quality and will be particularly useful for the future genetic improvement of tea plants.

## DNA methylation of tea plants

DNA methylation is among the most essential and ubiquitous epigenetic modifications in plants^115^. It plays crucial roles in gene expression regulation, cell differentiation, and transposon element (TE) silencing^116^. DNA methylation are mostly investigated in model plants, such as *Arabidopsis* and rice, due to their small genome sizes and low genome complexity^117,118^. Tea plants are nonmodel woody plants with complex nuclear genomes. The sequencing of the tea plant genome has revealed an extraordinary amplification of genome size driven primarily by a burst of TEs^1,2^. DNA methylation was confirmed to be closely linked with TE burst in tea plants^119^. Compared to asterids such as potato, tea plants exhibit higher CHG methylation levels, similar to those found in maize and *Norway spruce*, two plant species with a comparable genome size and repeat content to tea plants^120,121^. Remarkably, the DNA methylation levels of TEs vary with their evolutionary distance in tea plants, with the methylation level increasing in recent, active TEs and decreasing in ancient TEs. It is widely accepted that DNA methylation can spread from TE boundaries to close genomic regions, resulting in increased methylation levels of adjacent genes^122^. High TE contents in tea plants might therefore contribute to the high level of DNA methylation. Several genes involved in the biosynthesis of secondary metabolites (catechins, theanine, and caffeine) were found to be TE-related in tea plants. This suggests that TE-mediated DNA methylation may have some effects on the formation of tea quality.

Chilling damage caused by low temperature occurs commonly in tea plants and has severely affected the sustainable development of the global tea industry. Approximately 49–51% of cytosine residues are methylated during the cold acclimation of tea plants^123^. Compared to preacclimation, the DNA methylation level increases significantly during cold acclimation and is maintained at a higher level after deacclimation. The gene encoding DNA methyltransferase, *CsDRM2*, was cloned from an elite cultivar of tea plant (Longjing #43) and found to exhibit high expression levels under cold acclimation^124^. In the future, additional DNA methylation projects need to be performed in relation to other aspects of tea plants to elucidate the roles of these modifications in plant development, environmental adaptation, stress responses, and even genome structure variation and stabilization.

## ncRNAs of tea plants

MicroRNAs (miRNAs), a class of endogenous small ncRNAs, have been recognized as a critical genetic regulator primarily engaged in posttranscriptional regulation. In tea plants, a series of studies have been undertaken to examine the posttranscriptional regulation of miRNAs in development^125,126^, metabolism^126–128^, and responses to biotic/abiotic stresses^129–133^. Tea plant miRNAs can negatively regulate catechins and terpenoid biosynthesis by down-regulating target genes related to their biosynthesis at both the transcriptional and posttranscriptional levels^126,128^. For instance, Cs-miR156 regulates catechin accumulation in tea plants by suppressing the expression of the target gene SPL in the presence of different nitrogen forms^128^. The characterization of tea plant miRNA regulatory networks in response to *Colletotrichum gloeosporioides*, which is considered one of the dominant endophytic taxa in tea plants, indicated that miRNAs may be involved in the response to *C. gloeosporioides* attack^134^. miRNA characterization in tea plants under cold and drought stresses suggested the potential existence of an miRNA-mediated regulatory mechanism under abiotic stresses showing coherent or incoherent relationships with target genes to prevent tea plants from being injured^129–132^. Csn-miR398a-3p-1 of tea plants was experimentally proven to directly cleave *CsCSD4*, a superoxide dismutase (SOD) gene related to the removal of reactive oxygen species (ROS), and the expression pattern of csn-miR398-3p-1 is opposite that of *CsCSD4* under cold treatment^135^. Roles of miRNAs have also been found in the bud dormancy of tea plants. Cs-miR139c regulates the release of tea plants from bud dormancy by suppressing the expression of its target gene, *CsnTCP2* (Teosinte branched/Cycloidea/Proliferating cell factor 2)^136^. There is no doubt that the investigation of miRNAs provides a broad understanding of the posttranscriptional regulatory networks between protein-coding and ncRNAs; however, most current progress is limited to the identification, but not the functional verification, of miRNAs, which will require further investigation in tea plants.

In addition to miRNAs, long noncoding RNAs (lncRNAs) have been also identified in tea plants using 170 RNA-seq data (~7157 million reads) from 11 tissues, generating more than 33,000 putative novel lncRNAs^137^. The tea plant lncRNAs showed tissue-specific expression patterns and were suggested to be involved in major aroma formation pathways of black tea^137^. circRNAs (circular RNAs) were recently discovered as a new class of ncRNAs that are covalently closed, single-stranded and generated via back-splicing events^138,139^. Since their first documentation in *A. thaliana*^138^, circRNAs have been detected in several other plants^138,140–142^. A total of 3174 circRNAs have been characterized in tea plants^141^. As similarly observed in most other plants^139^, tea plant circRNAs exhibit tissue-specific expression patterns, and their expression shows a positive relationship with that of their parental genes^141^. Tea plant circRNAs were found to exhibit potential miRNA-binding sites and to display crucial functions in the photosynthetic machinery and metabolite biosynthesis during leaf development^141^. In other plants, circRNAs can act as miRNA sponges under tomato chilling injury^142^, dehydration stress in wheat^143^, and infection with the bacterial canker pathogen in kiwifruit^144^. These studies have increasingly complicated our understanding of ncRNAs and their regulatory mechanisms in tea plants. Additionally, none of these identified ncRNAs (miRNAs, lncRNAs, and circRNAs) have been functionally well-verified regarding their roles in regulating posttranscriptional processes in tea plants. Further efforts aimed at accurate lncRNA characterization and the functional examination of their interaction mechanisms in competing endogenous RNA (ceRNA) networks should receive more attention to elucidate their roles in response to stress and the regulation of tea plant secondary metabolite formation.

## Genetic diversity and population structure of tea plants

**Fig. 2:**
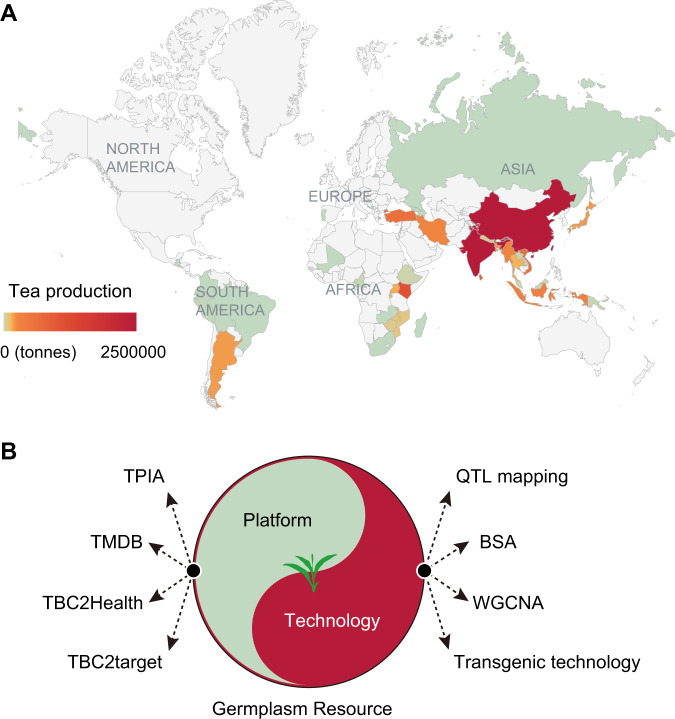
Global tea production and platforms/techniques for scientific studies of tea. **a** Global tea production across the majority of tea-producing countries. The data were collected from 2017 FAO statistics (http://www.fao.org/). **b** Resource-centered research platforms and technologies for tea molecular biology.

In the thousands of years since the tea plant was first discovered, recorded, and cultivated to produce tea in China, it has spread to more than 50 countries around the world in tropical and subtropical regions^1^ (Fig. 2a). The wide range of growing areas, long history of cultivation, self-incompatibility and allogamy of tea plants make these cultivars highly heterogeneous, resulting in high diversity in both their genetics and morphology^145,146^. As the key component of crop genetic improvement, the available germplasms and the genetic diversity within a gene pool determine the success of breeding programs. The development of molecular markers, such as RAPD, AFLP, SSR, and SNP markers, has allowed the successful utilization of these markers to estimate the genetic diversity and determine the phylogenetic relationships of different tea germplasms^147–153^. Early in 1995, the systematic assessment of the genetic variability of three major types (CSA, CSS, and Cambod type) of tea plants from Kenya and the UK suggested that the Cambod population possessed the lowest diversity^153^. The further investigation of genetic and morphological traits revealed consistent findings in populations from other major tea plantation countries^145,147,154–158^. Germplasm from the Korean population shows higher diversity than that from Japan due to more extensive plantations in Korea and longer, intensive tea selection programs in Japan^157^. The genetic diversity of the Chinese, Indian, and Sri Lankan populations is higher than those from other countries^151^. Most Italian tea plants exhibit a close relationship with cultivars from Zhejiang Province^159^. Kenyan tea plants show the highest diversity among all African germplasms, while the lowest is found in South Africa^160,161^. CSA was indicated to have donated the largest amount of genetic material in African tea breeding^161^. Narrow genetic diversity is detected in the Sri Lankan population, which is dominated by Assam-type tea^162^. The estimation of worldwide tea plant genetic diversity has increased our understanding of the population structures and origins of tea plants, which will benefit the global tea industry.

China hosts abundant tea plant germplasms. A wide range of genetic diversity and population studies have been undertaken, particularly for Chinese germplasms^148,149,152,163–167^. Most of the Chinese tea germplasms can be clustered based on their geographical origin and genetic background^149^. Higher levels of genetic diversity are observed in Guangxi, Yunnan, and Guizhou Provinces^148,167^. Compared to the wild tea plants, such as *C. taliensis*, the cultivated tea plants display higher heterozygosity^152^.

Molecular markers are useful and are commonly employed in crop breeding. Traditional experimental screening of marker polymorphism is laborious and time-consuming, especially for the broadly used SSR markers. The development of the CandiSSR pipeline has facilitated the efficient identification of polymorphic SSRs (PolySSRs) based on high-throughput sequencing data, and this pipeline has successfully identified 450 PolySSRs in the transcriptomes of four *Camellia* species^168^. Despite the diverse molecular and morphological markers developed, more efforts are still needed to develop extra-robust markers (e.g., SNPs) to better facilitate the conservation and utilization of global tea resources.

## Platforms and tools for tea plant functional genomics

### Databases

Biological databases provide scientists with an opportunity to centrally access a variety of biological data. Various tea plant databases have been established to help the tea-related research community to better understand the metabolome, health benefits, and genomics of tea^169–171^ (Fig. 2b). Yue and colleagues constructed the first comprehensive manually curated Tea Metabolome Database (TMDB)^171^, which contains over 1400 metabolites and 600 NMR datasets collected from 364 publications. Retrieval from the TMDB can thoroughly access a total of 24 basic attributes of tea compounds of interest, such as compound structure, molecular weight, and compound uses. The beneficial effects of tea are principally derived from diverse bioactive compounds. The establishment of the searchable TBC2health database has connected the relationships between tea compounds and their health beneficial effects well^169^. The current release of TBC2health compromises 497 bioactive tea compounds and their associated health effects on 206 diseases. The user-friendly response interface contains multiple easily visualized results, aiding in the efficient discovery of potential mechanisms of the health benefits of tea. Increasingly, studies have confirmed that the health benefits of tea are primarily achieved through the regulation of target gene expression or protein activities^172,173^. More recently, the TBC2target database was developed to provide candidate target genes for a total of 240 bioactive tea compounds^170^. The target genes were predicted using PharmMapper via a pharmacophore-mapping strategy^174^. With TBC2target in hand, scientists can explore the potential genes targeted by tea compounds and then reveal the possible health-promoting mechanisms of bioactive tea compounds.

Tea plants are rich in secondary metabolites that essentially determine tea quality^2^. Hundreds of studies on tea quality and physiology have been reported, which have generated a wide variety of specific biological data^1,2^. The recently developed Tea Plant Information Archive (TPIA) database employs the published tea plant genome as a basic framework and incorporates a wide range of publicly available genomic, transcriptomic, and metabolomic data of tea plants together with globally collected tea germplasms^175^. It also comprises over 70 transcriptomes widely collected from 21 *Camellia* species, plentiful gene expression data from various conditions (across species, stresses, and hormone treatments), orthologs, transcription factors, SSRs, metabolites (mainly catechins, theanine, and caffeine), correlations, and manually curated functional genes. Through long-term maintenance and timely updating with novel datasets, the TPIA is gradually becoming the central gateway for tea communities to investigate the biology of tea plants in more detail, thus benefitting the sustainable development of the global tea industry.

### Quantitative trait loci (QTL) mapping and bulked-segregant analysis (BSA)

QTL mapping is an efficient tool for revealing the molecular basis of complex traits of organisms^176^. Despite the fact that QTL mapping is widely used in several crops^177,178^, the application of this method to tea plants is still challenged, mainly due to the self-incompatible nature of tea plants, which exhibit a low seed yield, leading to the propagation of insufficient populations for QTL mapping. Nevertheless, considering the importance of tea plants in global tea consumption, increasing efforts are being focused on the QTL mapping of several agronomic traits of tea plants, particularly tea yield^20^ and secondary metabolites^22,27–29^ (Fig. 2b). A total of 23 putative QTLs associated with tea yield were detected using 5-year yield data from 42 F1 individuals collected across two sites in Kenya^20^. These efforts led to the first QTL-mapping investigation in tea plants, but there was no shared QTLs discovered at the two sites, indicating an overestimated QTL effect due to insufficient mapping populations. In contrast to yield traits, QTLs related to tea quality have been widely explored. Using 2-year catechins data from 183 individuals crossbred from two tea plant varieties with distinct catechins composition, a total of 25 catechin-related QTLs were identified^27^. Nine of these QTLs were validated across years and found to cluster together in the chromosomal regions of LG03 and LG11, suggesting a potential tandem duplication origin of tea plant catechin genes. Similarly, 10 QTLs were found to be involved in the determination of theobromine and caffeine contents using 148 individuals from a pseudo-testcross population^22^. With the exception of one QTL related to caffeine content, which was validated across years and mapped on LG01, the other QTLs were validated from only 1 or 2 years of data, possibly due to the relatively small size of the mapping populations. With the expansion of mapping populations, a recent QTL mapping study detected several novel QTLs related to flavonoids^29^. Among the 27 identified QTLs associated with flavonoids, 7 were newly revealed to be involved in the determination of anthocyanin content and young shoot color. Interestingly, quite different from other recent reports^22^, QTLs controlling caffeine content were identified on LG11 in this study^29^. This implies that the caffeine content of tea plants is probably controlled by multiple genes located on different chromosomes or that the QTL effects have been overestimated because of the limitations of the mapping populations. In addition to tea quality and yield traits, QTL mapping has been applied to agronomic traits such as spring bud flush and leaf size (e.g., mature leaf length, width, and shape indexes)^28^. In the future, efforts will be needed to further increase the mapping populations and improve the density of genetic linkage maps to precisely identify QTLs in tea plants.

Although over 80 QTLs have been detected for diverse agronomic traits of tea plants, it is still difficult to explore the candidate genes and/or alleles related to QTLs further owing to the insufficient genetic markers and genomic information available. The recently developed BSA method selects and pools samples from individuals with extreme phenotypes from biparental segregation populations and enables the efficient identification of genes and alleles governing complex traits through statistical genomic and phenotypic analyses. In tea plants, the utilization of the BSA method coupled with RNA sequencing has facilitated the discovery of a flavonoid 3′,5′-hydroxylase (*F3*′*5*′*H*) gene that is significantly associated with catechin content based on individuals from the two tails of an F1 population that segregates with catechin content^179^.

### Weighted correlation network analysis (WGCNA)

WGCNA is of particular importance for tea plants to establish the relationships between gene expression and secondary metabolites that determine tea quality^180,181^ (Fig. 2b). It is also helpful for identifying clusters of highly correlated genes and revealing their regulatory networks in tea plant development^182,183^. The investigation of the expression profiles of tea plants at 10 developmental stages identified a total of 35 coexpression modules using the WGCNA method with differentially expressed genes (DEGs)^182^. Among these, 20 modules were found to be related to the biosynthesis of catechins, theanine, and caffeine, which were biologically coregulated by several hub genes, suggesting a potential network of coordinated regulation in three characteristic secondary metabolic pathways of tea plants. Building the association between tea aroma accumulation and gene expression using the WGCNA method remarkably revealed 13 transcription factor genes that are probably involved in terpene metabolism^181^. Among these genes, *CsOCS2* was validated in vitro to function in terpenoid biosynthesis, indicating a robustness and reliability of WGCNA for trait-associated gene discovery. Similarly, the application of WGCNA identified a gene module consisting of eight genes significantly associated with the biosynthesis of cyanidin 3-O-glucoside and petunidin 3-O-glucoside, helping to elucidate the mechanism of pigmentation in the anthocyanin-rich tea plants^180^. These findings suggested that, compared to several previous correlation studies^1,2,60^, WGCNA is much more powerful; however, the sample sizes and/or traits used in most of the current studies are still limited, which decreases the accuracy and scope of the acquired candidates. The integration of more samples and traits from diverse cultivars and conditions in the future will improve the capability of WGCNA and thus enhance the rapid discovery of candidate genes associated with important agronomic traits in tea plants.

## Future challenges and perspectives

### Completion of the tea plant genome with advanced technologies

**Fig. 3:**
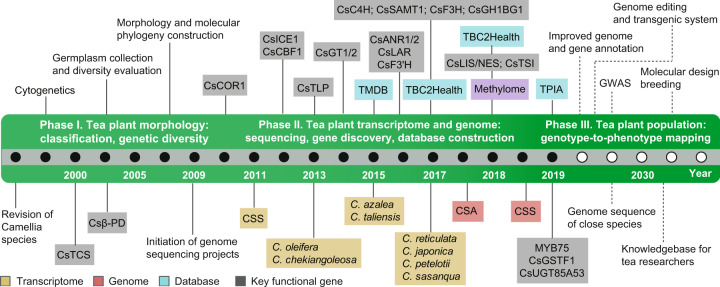
Timeline of research on tea plant genetics and genomics. The solid black circles indicate past events in tea plant genomics, including Phase I of tea plant morphology and Phase II of tea plant transcriptome and genome studies. Events are highlighted with colored rectangular boxes: yellow (transcriptome sequencing), orange (genome sequencing), cyan (database development), and gray (gene cloning). The solid white circles represent Phase III of tea plant comparative genomics and population genetics studies in the future.

Tea originated from China and has spread to over 160 countries around the world. According to statistics from 2017 from the Food and Agriculture Organization of the United Nations (FAO; http://www.fao.org/), the global tea plantation area has exceeded 4.08 million hectares, and global total tea production has reached 6.10 million tons, with an average increase of 0.33 million hectares and 0.58 million tons over the past 5 years (Fig. 2a). In stark contrast to the flourishing development of the global tea industry, research on the major fundamental biological problems encountered in tea plants is still lagging behind, resulting in a low breeding efficiency and few excellent varieties. The rapid development and application of genomics approaches have effectively promoted the breeding programs of crops; however, genomic investigations of tea plants are still limited and challenging. Although the genome sequences of two major varieties (CSS and CSA) of tea plants were generated recently, their quality and completeness still need to be improved^1,2^. Given the limitations of current NGS-based approaches related to highly repetitive and heterozygous genomes, advanced single-molecule-sequencing technologies (e.g., PacBio SMRT and Oxford Nanopore) will help resolve the highly repetitive regions of the tea plant genome. With the further inclusion of linkage map and Hi-C technology to interactively address heterozygous genomic regions, the eventually produced chromosome-level reference genome of tea plants will facilitate comparative genomics, evolutionary and population genetics studies and, thus, promote breeding programs in the future (Fig. 3).

### Towards tea plant pan-genomics and the phylogeny of genus *Camellia*

Tea plants are widely distributed in tropical and subtropical regions around the world^184^. They contain rich and unique characteristic secondary metabolites, such as catechins, theanine, and caffeine, which are essential to the formation of tea quality. However, the contents of these secondary metabolites vary greatly among different varieties and *Camellia* species, leading to large differences in tea processing suitability^1,185^. They also differ significantly in several morphological traits (e.g., leaf size) and stress resistance characteristics (e.g., cold tolerance), showing a divergent genetic makeup^50,186^. Therefore, the genome sequence of a single individual of a tea plant variety (e.g., shuchazao or yunkang #10) cannot represent the entire gene pool of tea plants. Pan-genome sequencing is a newly developed strategy for investigating the genetic variations among different subspecies and/or individuals that has been widely used in several crops and model species, such as rice^187^, soybean^188^, maize^189^, and *Arabidopsis*^190^. With the completion of the tea plant genome, more efforts are still needed to further investigate the pan-genomics of tea plants from different varieties and/or ecotypes to expand the tea gene pool. However, the phylogeny of genus *Camellia* and the relationships among different tea populations remain largely unclear, which has seriously hindered the in-depth exploration of pan-genomics in tea plants.

Although several studies have attempted to resolve the phylogeny of genus *Camellia* using chloroplast and/or mitochondrial DNA sequences, the generated phylogenetic trees are somewhat poorly supported, primarily due to the high frequency of hybridization and polyploidization, as well as the relative conservation of organelle DNA sequences among *Camellia* species^39,45,191–193^. Compared to organelle fragments, massive nuclear gene sequences rapidly obtained from genome and transcriptome sequencing provide abundant candidate low-copy-number nuclear genes and informative sites for phylogenetic investigation and have been increasingly applied to clarify the phylogenies of some complex plant taxa^194–198^. Therefore, the sequencing of additional genomes and/or transcriptomes of *Camellia* species, as well as elite tea cultivars in the foreseeable future will not only help to reveal their complex phylogenetic relationships but will in turn promote pan-genomic studies of tea plants to expand the gene pool.

### Genome-wide association study (GWAS) of tea plants

GWAS is a widely accepted strategy to identify genes/alleles associated with complex traits in crops with the rapid innovation of NGS technologies^199–202^. Genotypes (typically based on SNPs) and phenotypes (usually those of different traits) are the two fundamental requirements in GWAS, in which a higher SNP density and more diverse phenotypes of a population will theoretically produce more reliable and significant associations. However, conventional GWAS design and methods have resulted in challenges in the identification of multiple functional alleles within a single gene as well as rare alleles in the population^203,204^. GWAS of tea plants has also been confronted with major difficulties in population construction and phenotyping related to germplasm collection and the ultraslow growth of tea plants when transplanted to nursery gardens. Nevertheless, considering the high level of genetic and morphological diversity in tea plants, GWAS is still the most efficient way to detect candidate loci of complex traits undergoing improvement. To apply GWAS to study the genetics underlying tea traits of interest, the following goals should be considered: (1) collection and construction of core germplasms with adequate genetic diversity; (2) transplantation of the germplasms to one or more local gardens for accurate multiple-year phenotyping; (3) further improvement of the quality and annotation accuracy of the current genome assembly; (4) integration of GWAS data with other omics datasets (e.g., transcriptomic and metabolomics data); and (5) construction of an efficient transgenic system to functionally validate the candidates. In this way, GWAS can be effectively applied to understand the genetic basis of agronomic traits associated with tea quality, thus enabling the molecular design of new cultivars in the near future.

### Origin and domestication of tea plants

China is documented as the first country to cultivate and utilize tea plants in the world^205^. It harbors abundant tea germplasms and has long been considered the origin of tea plants^206^. However, since no wild ancestor of tea plants has been discovered in China, the origin and domestication of tea plants are still a mystery and attract worldwide attention. Although recent progress has been made in understanding the origin and domestication of tea plants based on molecular markers (primarily SSRs), the resultant conclusions are unilateral and even controversial due to the poor representativeness of sample collections and the lack of sufficient molecular markers for diversity evaluation^160,161,207–211^. The recent release of tea plant genome sequences has laid a solid foundation for solving this problem^1,2^; however, further efforts focusing on the following issues are still needed: (1) collection of globally representative tea plant samples; (2) investigation of the population structure of tea plants and identification of their putative wild ancestor; (3) estimation of population diversity using genome-wide SNP markers; (4) scanning of genomic regions with significantly lower diversity in cultivated but not wild tea plants and identification of the candidate genomic regions selected during domestication; and (5) functional investigation of the characteristics of genes within regions of domestication. It should be noted that most of the existing tea varieties are bred and propagated directly from natural populations. Artificial domestication may have had little impact on the variation in genome sequences. Therefore, tea plants may actually undergo adaptive evolution compared to other crops (e.g., rice) subjected to strong domestication pressure^212^.

### Establishment of high-efficient transgenic system

*Agrobacterium*-mediated genetic transformation has been extensively applied to verify the function of genes with the aim of improving the quality and yield of crops. In tea plants, the first transgenic plant was generated using *Agrobacterium*-mediated transformation in somatic embryos^213^. However, at least 45 weeks were possibly needed for the transformation procedure alone, and additional years were required for further transplantation^213^. It also required several years of experimental work to establish stability and germline transmission in these plants. Although several efforts have been made to optimize the transformation system of tea plants using different strategies^214–223^, transformation efficiency still represents a challenge under the available methods^215,224^. In the face of these challenges, some key points related to transgenic technology need to be further detailed: (1) optimization of the transformation and regeneration system; (2) clarification of the transformation mechanism of tea plants, particularly to elucidate the effects of tea polyphenols on *Agrobacterium* infection; and (3) identification of more suitable tea varieties for better transformation and regeneration. The most recent CRISPR system has enabled efficient genome editing in crops and shows great potential for accelerating precise crop improvement^225^. Considering the difficulties encountered transgenic studies of tea plant, the establishment of the CRISPR system will promote the transgenic progress in tea plants^221^. More recently, scientists demonstrated an efficient approach to overcome self-incompatibility in diploid potato using the CRISPR/Cas9 system through *S-RNase* locus knockout, which provided a useful example for the further application of transgenic technology and breeding in tea plants, considering their similar self-incompatibility features^226^.

### Collection and conservation of tea plant germplasms

Resources are clearly the key to crop genetic improvement. The success of plant breeding and conservation is largely dependent on the amount and distribution of genetic variations present in collections. Currently, tea germplasms are becoming the most valuable fundamental material for tea breeding and biotechnology studies, presenting great potential in the whole tea industry^227^. The remarkable achievements made in worldwide tea plant germplasm surveys, collection, and conservation have preserved more than 15,000 tea plant accessions according to incomplete statistics from the China Tea Science Society (http://www.chinatss.cn/). These invaluable germplasms have driven the extraordinarily rapid development of tea plant genomics, genetics, and breeding^151,152,175,207,228,229^. However, most of the current accessions that have been collected and used are varieties, and few “wild” or close relatives have been integrated. Another major problem that is currently faced by the field is the imbalanced utilization and protection of cultivars versus “wild” germplasms. The survival environment of “wild” tea resources has been constantly destroyed with rapid economic development and urbanization. In addition, local germplasms (landraces) with specific characteristics are on the edge of being lost due to the popularization of elite varieties in the past few years. Above all, the surveying, collection, and conservation of tea plant germplasms should be urgently emphasized. Otherwise, no diverse tea plant resources or only a few mono varieties will survive and be available for utilization for the genetic improvement of tea in the future. We therefore propose that a tea plant conservation alliance uniting the global tea-planting countries needs to be established to accelerate the preservation and utilization of global tea plant resources.

### In-depth understanding of the complex secondary metabolism network

The overall importance of tea plants as an economic crop is due to the health-promoting functions of teas as non-alcoholic drinks that are popular worldwide^230^. Numerous studies have documented the health benefits of various tea drinks in humans, and studies continue to produce more fundamental discoveries about the effects of functional tea components on the improvement of human heath^231,232^. It is not only the three major types of tea plant secondary metabolites (catechins, caffeine, and theanine) but also the volatile terpenoids, saponins, polysaccharides, and other phenolic conjugates found in tea that contribute to the beneficial health effects and the enjoyable flavors of various teas^70,71,233^. As mentioned above, our understanding of these complicated secondary metabolites in tea plants in terms of their biosynthesis, transport, and storage as well as their regulation is very limited. This is one of the major obstacles to the breeding of high-quality tea plant varieties with biofortified nutrients or functional components or other special properties. Future work could focus on basic questions about tea secondary metabolism, which will facilitate the breeding of tea plants with desirable qualities and benefit human health improvement projects.

In conclusion, tea plants are perennial woody plants in nature with a long growth cycle, which severely limits their genetic breeding. Traditional cross-breeding is extremely difficult and time-consuming for tea plants because most of the existing tea varieties are bred and propagated directly from natural populations. Modern transgenic breeding technology has provided us a new solution for the molecular design of breeding strategies, which basically relies on the development of functional genomics and molecular biology. Although great progress has been made in the last two decades, the genomics and molecular biology of tea plants are still not fully understood, mainly due to difficulties in resource collection and identification, the generation and identification of mutant plants, and population construction. In particular, the limited knowledge of the functional genomics and developmental biology of tea plants has narrowed our understanding of their basic biological characteristics. Therefore, compared to other crops such as rice, there is still a long way to go in tea plant genomics and molecular biology research. In the near future, scientists should focus more on industry-oriented major basic biological research based on germplasm collection and utilization, particularly by deciphering the tea plant genome, as an opportunity for enhancing studies on the mechanisms of the biosynthesis and regulation of secondary metabolites, the genetic basis of important agronomic traits, the molecular mechanisms of stress and disease resistance, and the developmental biology of tea plants, to accelerate the molecular design breeding program and, thus, promote industrial development.

## Supplementary information

Supplemental Table 1

Supplemental Table 2
